# 浆母细胞淋巴瘤诊断与治疗中国专家共识（2025年版）

**DOI:** 10.3760/cma.j.cn121090-20241213-00566

**Published:** 2025-04

**Authors:** 中华医学会血液学分会淋巴细胞疾病学组 中国临床肿瘤学会（CSCO）淋巴瘤专家委员会

## Abstract

浆母细胞淋巴瘤（plasmablastic lymphoma，PBL）起源于生发中心终末分化期的活化B细胞，具有独特的临床、病理及分子学特征，呈现高度侵袭性、预后较差。PBL发病率低，诊断具有挑战性，治疗缺乏规范性。为加强我国临床医师对PBL的认识，提高诊断及治疗水平，有助于推动开展多中心临床研究，中华医学会血液学分会淋巴细胞疾病学组、中国临床肿瘤学会（CSCO）淋巴瘤专家委员会组织相关专家，讨论并形成本共识。

## Abstract (en)

Plasmablastic lymphoma（PBL）originates from activated B cells in the terminal differentiation stage of the germinal centers. PBL is characterized by unique clinical, pathological, and molecular features, demonstrating high aggressiveness and poor prognosis. It exhibits a low incidence and a challenging diagnosis, with a lack of standardized treatment protocols. To improve the understanding of PBL among clinical physicians in China and enhance diagnostic and treatment levels, which will facilitate the initiation of multicenter clinical research, the Lymphocyte Disease Group of the Hematology Division of the Chinese Medical Association and the Lymphoma Expert Committee of the Chinese Society of Clinical Oncology（CSCO）have organized relevant experts to formulate this consensus.

浆母细胞淋巴瘤（plasmablastic lymphoma，PBL）属于高度侵袭性非霍奇金淋巴瘤，呈现免疫母细胞和浆母细胞形态，具有浆细胞免疫表型特征，预后较差。PBL发病率低，诊断具有挑战性，治疗缺乏规范性。为加强我国临床医师对PBL的认识，提高诊断及治疗水平，推动开展多中心临床研究，中华医学会血液学分会淋巴细胞疾病学组、中国临床肿瘤学会（CSCO）淋巴瘤专家委员会组织相关专家，在系统总结国内病例资料基础上，参考国外相关临床实践指南[1]–[2]，讨论并形成本共识。

一、概述

PBL于1997年首次被报道，93.7％（15/16）为人类免疫缺陷病毒（human immunodeficiency virus，HIV）阳性患者[3]，初发病灶均来自口腔部位。其他报道也可见口腔以外部位。除HIV感染外，PBL还可发生于其他原因导致免疫功能缺陷的患者中，如器官移植术后、自身免疫性疾病等。但后期研究发现部分患者并无明确引起免疫功能缺陷的基础疾病[4]–[5]。国外资料[6]显示，PBL的年发病率约0.07/10万人，中位发病年龄约57岁，男女比例约3∶1，HIV阳性患者发病年龄小于HIV阴性患者[7]。PBL具有独特的细胞形态及免疫表型，侵袭性强。2008年世界卫生组织造血与淋巴组织肿瘤分类将其列为大B细胞淋巴瘤的一个独立亚型并保留至今。PBL占大B细胞淋巴瘤比例约0.7％，占HIV感染相关淋巴瘤的比例约为2％[8]。随着诊断水平提高及多药联合治疗，PBL患者预后得到极大改善，国际报道的中位总生存（OS）期为32～58个月[8]。国内报道病例中HIV阳性比例不足5％，男女比例约3∶1，中位发病年龄与国外相似，2年OS率约为65.2％[5]。

二、发病机制

PBL起源于生发中心终末分化期的活化B细胞，经历体细胞高频突变和免疫球蛋白（Ig）的类别转换，处于浆母细胞发育转化成浆细胞阶段。在这过程中，内在遗传性改变和外在病毒感染共同促使细胞发生恶性转化。约70％以上PBL患者存在MYC蛋白过表达，从而突破B淋巴细胞诱导成熟蛋白1（B lymphocyte-induced maturation protein，BLIMP1）的抑制作用，进而阻碍B细胞向浆细胞的分化。此外，JAK-STAT、ERK-MAPK和NOTCH信号通路相关分子突变亦常见，发挥协同致病作用[9]–[10]。EB病毒（Epstein-Barr virus，EBV）通过抗原相关的多种机制阻止B细胞凋亡，其在PBL致病中起着重要作用[11]。与EBV阳性患者相比，EBV阴性的PBL患者具有更高的遗传不稳定性和突变负荷，多涉及TP53、CARD11及细胞周期相关基因等突变，预后相对更差[12]。

三、临床表现

通常以进行性增大的无痛性肿物或肿物压迫周围组织或器官所致的相应症状为首发表现，任何淋巴结或结外部位都可能累及。国外报道[7]HIV阳性PBL患者最常见受累部位为口腔（48％），其次为胃肠道（12％）、皮肤（6％）和淋巴结（5％）等，而HIV阴性PBL患者常见受累部位为口腔（40％）、胃肠道（21％）和淋巴结（9％）等，器官移植术后PBL患者则以淋巴结累及多见（30％），其次为皮肤（22％）。

国内报道[13]HIV阳性PBL患者多以口腔颌面部起病，常伴有结外部位累及，而HIV阴性PBL患者则以淋巴结、胃肠道受累多见，分别约占20％，其次为鼻腔、口腔等部位[4],[14]，中枢神经系统[15]、软组织[16]和骨[17]等均可累及，与国外报道存在一定的差异。初诊时分期为晚期患者约占60％、骨髓受累约占30％、B症状常见。

四、诊断及鉴别诊断

（一）病理诊断

1. 病理取材：通过病灶部位的病理活检进行确诊，优先推荐病变部位切取活检；对于深部或腔道器官病变，空芯针穿刺或经内镜活检也是可行方式，前提是尽可能获取足够组织标本用于诊断及鉴别诊断。

2. 形态学特点：肿瘤细胞弥漫成片破坏性浸润，呈免疫母细胞、浆母细胞形态，核分裂象易见，可见凋亡小体及由巨噬细胞吞噬核碎片而形成的“星空”现象。在罕见情况下，肿瘤细胞少数可呈中等大小的淋巴浆细胞样和浆细胞样形态。肿瘤细胞形态与病变部位、HIV感染情况等存在相关性，HIV阳性和病变部位为口腔、鼻腔和副鼻窦区的患者，其肿瘤细胞更多表现为单一浆母细胞形态，而病变部位为其他结外部位和淋巴结等的患者，其肿瘤细胞更多呈浆细胞样形态[18]。

3. 免疫表型：PBL肿瘤细胞CD20表达缺失，PAX5和CD45（LCA）表达减少或缺失。CD79a在约40％的病例中呈阳性。大多数情况下，肿瘤细胞表达浆细胞分化相关的标志物，如CD138、CD38、VS38c、BLIMP1和XBP1。IRF4（MUM1）大多数阳性[3],[19]–[21]，常表达IgG，Kappa、Lambda轻链限制性表达。Ki-67增殖指数>90％[20],[22]。PDL1过表达[21],[23]，CD10、CD56和CD30在20％～30％的病例中呈阳性，而BCL6较少阳性。约60％的病例EBV阳性[9]，且更常见于HIV阳性PBL患者（82％）。ALK和人疱疹病毒8型（human herpesvirus 8，HHV-8）潜伏相关核抗原（LANA）均为阴性。罕见异常表达胞质CD3[24]、CD43、CD45RO等T细胞标志物，<10％的病例异常表达细胞角蛋白，需与（未分化）癌进行鉴别诊断。

（二）鉴别诊断

需结合临床特征、免疫表型和病毒学检查，必要时可借助分子学检测手段进行鉴别诊断。其中浆细胞骨髓瘤（plasma cell myeloma，PCM）、慢性炎症相关弥漫大B细胞淋巴瘤（DLBCL-CI）和原发性渗出性淋巴瘤（primary effusion lymphoma，PEL）主要借助临床特征和PBL进行鉴别；而DLBCL非特指型（免疫母细胞亚型）［DLBCL-NOS（IB）］和DLBCL-CI则主要依赖免疫表型特点与PBL区分；HHV-8阳性弥漫大B细胞淋巴瘤（HHV-8^+^ DLBCL）和PEL患者常合并HHV-8相关感染，可与PBL进行鉴别；而间变性淋巴瘤激酶阳性大B细胞淋巴瘤（anaplastic lymphoma kinase-positive large B-cell lymphoma，ALK^+^ LBCL）多存在ALK基因易位，可与PBL进行鉴别。具体鉴别要点可参考表1。

**表1:** 浆母细胞淋巴瘤（PBL）鉴别诊断要点

鉴别诊断要点	PBL	PCM	DLBCL-CI	DLBCL-NOS（IB）	HHV-8^+^ DLBCL	PEL	ALK^+^ LBCL
临床特征	口腔、胃肠道等	骨髓瘤相关的器官功能损害；M蛋白阳性	存在长期慢性炎症；胸腔多见，形成肿块	淋巴结累及多见	淋巴结累及多见	体腔恶性渗出且多数不伴肿块	淋巴结累及多见
病毒感染	HIV感染（70％）；EBV感染，潜伏0/Ⅰ型	无	EBV常见；潜伏Ⅲ型	无	HHV-8常见	HHV-8常见；多合并HIV、EBV感染，潜伏Ⅰ型	无
细胞表型	CD38^+^/CD138^+^/MUM1^+^；CD20^-^/PAX-5^-^	CD38^+^/CD138^+^；CD20^-^/PAX-5^-^	大多数CD20^+^/CD79a^+^；少部分CD38^+^/MUM1^+^；个别表达T细胞标志	CD20^+^/PAX-5^+^；CD38^-^/CD138^-^	CD20^+/-^，CD38^+/-^；CD138^-^/CD79a^-^	CD45^+^/CD38^+^/CD138^+^；CD19^-^/CD20^-^/CD79a^-^/PAX-5^-^	CD38^+^/CD138^+^；CD20^-^/CD79a^-^/PAX-5^-^
胞浆Ig表达	50％～70％轻链限制性表达	>90％	罕见	罕见	IgM/Lammda	罕见	IgA/轻链限制性表达
分子学特征	MYC基因重排/突变	PCM相关遗传性改变	TP53突变（70％）	MYC基因重排/突变	IgH/L未发生重排	MYC基因重排/突变少见；IgH/L发生重排	t（2;17）/t（2;5）

**注** PCM：浆细胞骨髓瘤；DLBCL-CI：慢性炎症相关弥漫大B细胞淋巴瘤；DLBCL-NOS（IB）：弥漫大B细胞淋巴瘤非特指型（免疫母细胞亚型）；HHV-8^+^ DLBCL：人疱疹病毒8型阳性弥漫大B细胞淋巴瘤；PEL：原发性渗出性淋巴瘤；ALK^+^ LBCL：间变性淋巴瘤激酶阳性大B细胞淋巴瘤；HIV：人类免疫缺陷病毒；EBV：EB病毒；IgH/L：免疫球蛋白重链/轻链

五、临床分期和预后评估

建议采用^18^F-氟代脱氧葡萄糖（^18^F-FDG）PET-CT作为初诊PBL患者分期的检查手段，推荐使用2014年Lugano修订版Ann Arbor分期系统[25]。国外多项回顾性研究[26]–[27]均提示国际预后指数（IPI）系统对PBL患者预后判断仍具有一定意义，评分>2分患者预后较差，主要与美国东部肿瘤协作组评分、分期相关。此外，与不良预后相关的因素还包括EBV阴性、HIV阴性及诱导治疗未获得完全缓解（CR）等[28]–[30]。

六、治疗

为规范PBL的治疗，本共识建议按图1流程进行治疗。

**图1:**
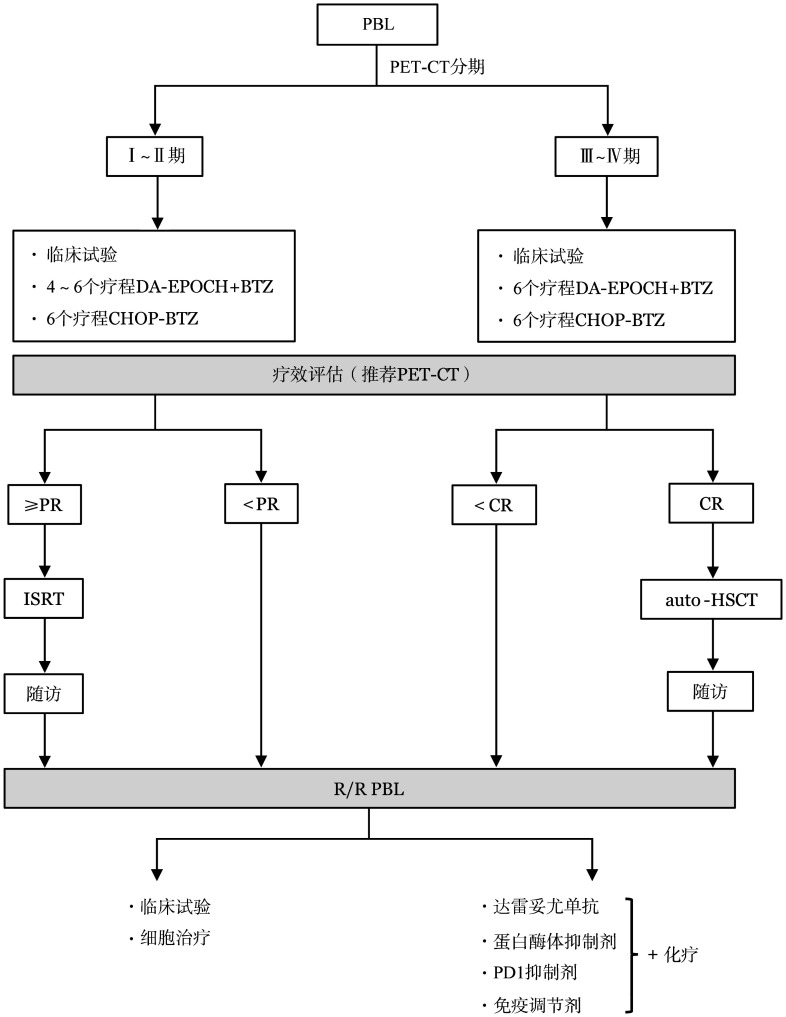
浆母细胞瘤（PBL）治疗流程图 **注** PET-CT：正电子发射断层扫描/X射线计算机断层成像；DA-EPOCH：环磷酰胺+表柔比星+依托泊苷+长春新碱+泼尼松+利妥昔单抗；BTZ：硼替佐米；CHOP：环磷酰胺+多柔比星+长春新碱+泼尼松；PR：部分缓解；CR：完全缓解；ISRT：受累部位放疗；auto-HSCT：自体造血干细胞移植；R/R：难治/复发

（一）治疗前评估

治疗前应对患者进行全面评估，应至少包括：

（1）病史和体格检查、体能状态评分；

（2）血常规、生化、乳酸脱氢酶、β_2_微球蛋白及M蛋白等；

（3）病毒学检测，包括HIV、EBV、HHV-8等；

（4）影像学检查：优先推荐全身^18^F-FDG PET-CT，其次选择颈、胸及全腹部增强CT、颅脑磁共振成像（MRI）、心脏彩超（左室射血分数等）；

（5）骨髓检查：细胞形态学、免疫表型及活检病理等。

（二）初治患者治疗原则

目前治疗策略依据多以回顾性研究为主，因此优先推荐参加临床试验。对于HIV阳性PBL患者，先行抗病毒治疗会导致患者病变部位肿块短期内迅速增大，加剧病情恶化[31]，目前多推荐同步或晚于抗肿瘤治疗。尽管有单独给予抗病毒治疗后肿瘤自行消退的病例报道，但短期内疾病即复发，故不建议单独使用。抗病毒治疗及疗效监测参考感染科会诊建议。

初诊时分期是影响PBL患者预后的重要因素，局限期患者预后较好。本共识建议依据临床分期进行个体化分层治疗，包括诱导和巩固治疗两个阶段。

诱导治疗方案选择参照侵袭性B细胞淋巴瘤，但既往以CHOP/CHOP样（环磷酰胺+多柔比星+长春新碱+泼尼松）方案为主的治疗CR率低，患者中位OS期不足1年。早期一项回顾性研究分析治疗强度对HIV感染相关淋巴瘤患者预后的影响，其中包括6％的PBL患者，结果表明较强的治疗策略如EPOCH（环磷酰胺+多柔比星+依托泊苷+长春新碱+泼尼松）方案等可延长患者生存时间，改善预后[32]。近期另一项针对HIV阴性PBL患者的预后分析显示，接受化疗233例，其中接受CHOP/CHOP样和其他较强治疗方案（如EPOCH等）的患者比例分别为53.2％（124/233）和18.9％（44/233），中位OS期分别为未达到和23个月，显示较强的治疗策略并未改善HIV阴性PBL患者预后[33]，可能与HIV阴性PBL患者年龄较大、耐受性差相关。因此，在明确老年患者治疗决策前需充分考虑疗效与安全性的平衡，建议采用老年综合体系评估，具体可参考《老年弥漫大B细胞淋巴瘤诊断与治疗中国专家共识（2024年版）》[34]。

CHOP/CHOP样方案联合用于治疗浆细胞肿瘤的新型靶向药物，如蛋白酶体抑制剂、免疫调节剂或抗体类药物为近年探索的方向[35]–[37]。多项回顾性小样本研究均证实硼替佐米（bortezomib，BTZ）联合EPOCH方案治疗PBL取得较好的近远期疗效[38]–[39]。其中一项研究纳入16例PBL患者，38％（6/16）为HIV阳性，接受蛋白酶体抑制剂联合EPOCH方案作为一线治疗，总反应率为100％、CR率为94％（15/16）、5年OS率为63％[39]。因此，本共识推荐诱导治疗采取CHOP/EPOCH+BTZ方案。

分层治疗主要体现在巩固治疗，一项国外多中心回顾性研究纳入1990年至2018年期间诊断为局限期且接受治疗的80例PBL患者，其中HIV阴性占79.5％，结果显示化疗序贯放疗3年无进展生存率和OS率分别为85％和96％，优于单纯化疗组（65％和71％）[40]。另一项研究针对局限期患者则予以BTZ+EPOCH方案治疗4次，序贯局部放疗，剂量为30～36 Gy，CR率和5年OS率均为100％[39]。另一项研究依据临床分期进行分层巩固治疗，患者局限期采取局部放疗，而进展期选择自体造血干细胞移植（auto-HSCT），中位随访48个月，患者总体5年OS率为63％[39]。因此，本共识建议局限期患者获得部分缓解以上疗效后予以局部放疗巩固，而进展期患者获得CR后行auto-HSCT巩固。

PBL易累及中枢神经系统，因此初诊时建议颅脑MRI评估，必要时进一步行腰椎穿刺相应检查明确有无中枢神经系统累及。对于初诊无中枢神经系统累及患者，建议常规采取大剂量甲氨蝶呤和（或）鞘内注射等进行中枢神经系统预防。

（三）难治/复发患者的治疗

难治/复发患者预后极差，中位OS期不足1年。治疗前尽可能再次活检明确疾病性质，同时行高通量基因组测序，探索个体化治疗策略[41]。难治/复发患者治疗优先推荐参加临床试验。对于无法参加临床试验的患者，可供参考的治疗方案（多为个案报道）如下：①达雷妥尤单抗联合ICE（异环磷酰胺+卡铂+依托泊苷）、DHAP（地塞米松+阿糖胞苷+顺铂）方案等[42]–[43]；②PBL细胞大多数表达BCMA[44]，BCMA嵌合抗原受体T细胞治疗难治/复发PBL也显示出较好的疗效[45]；③约70％ EBV阳性患者表达CD30和PDL1[46]，维布妥昔单抗、PD1抑制剂等联合其他治疗方案均有病例报道，如替雷利珠单抗联合来那度胺[47]等；④塞利尼索联合化疗等[48]。建议一线未接受移植的患者再次诱导治疗达到CR后可以选择auto-HSCT巩固。

七、疗效评估和随访

疗效评价标准参照Lugano 2014标准[25]，建议采用^18^F-FDG PET-CT进行疗效评价。完成治疗后的前2年应每3个月进行1次随访，并进行包括颈部、胸部及全腹部CT等检查。完成治疗后第3年至第5年每半年进行1次随访，每12个月进行1次CT检查。

八、总结

PBL具有独特的临床、病理及分子学特征，诊断需结合临床特征、免疫表型和病毒学检查等，必要时可借助分子学检测手段进行鉴别。随着新药的一线使用，PBL患者预后得到极大改善，但仍不尽人意，因此优先建议参加临床试验。初诊患者依据临床分期进行个体化分层治疗，制定治疗决策前需充分考虑疗效与安全性的平衡；难治/复发患者治疗存在瓶颈，目前探索的新型药物试图改善其预后。
